# Inhibition of phosphodiesterase 4 reduces ethanol intake and preference in C57BL/6J mice

**DOI:** 10.3389/fnins.2014.00129

**Published:** 2014-05-27

**Authors:** Yuri A. Blednov, Jillian M. Benavidez, Mendy Black, R. Adron Harris

**Affiliations:** Waggoner Center for Alcohol and Addiction Research, The University of Texas at AustinAustin, TX, USA

**Keywords:** alcohol, two-bottle choice, PDE inhibitors, rolipram, mesopram, piclamilast, CDP840

## Abstract

Some anti-inflammatory medications reduce alcohol consumption in rodent models. Inhibition of phosphodiesterases (PDE) increases cAMP and reduces inflammatory signaling. Rolipram, an inhibitor of PDE4, markedly reduced ethanol intake and preference in mice and reduced ethanol seeking and consumption in alcohol-preferring fawn-hooded rats (Hu et al., 2011; Wen et al., 2012). To determine if these effects were specific for PDE4, we compared nine PDE inhibitors with different subtype selectivity: propentofylline (nonspecific), vinpocetine (PDE1), olprinone, milrinone (PDE3), zaprinast (PDE5), rolipram, mesopram, piclamilast, and CDP840 (PDE4). Alcohol intake was measured in C57BL/6J male mice using 24-h two-bottle choice and two-bottle choice with limited (3-h) access to alcohol. Only the selective PDE4 inhibitors reduced ethanol intake and preference in the 24-h two-bottle choice test. For rolipram, piclamilast, and CDP840, this effect was observed after the first 6 h but not after the next 18 h. Mesopram, however, produced a long-lasting reduction of ethanol intake and preference. In the limited access test, rolipram, piclamilast, and mesopram reduced ethanol consumption and total fluid intake and did not change preference for ethanol, whereas CDP840 reduced both consumption and preference without altering total fluid intake. Our results provide novel evidence for a selective role of PDE4 in regulating ethanol drinking in mice. We suggest that inhibition of PDE4 may be an unexplored target for medication development to reduce excessive alcohol consumption.

## Introduction

Alcoholism is one of the most expensive (more than $185 billion/year in the US) and damaging chronic diseases (Bouchery et al., 2011), yet there are only three FDA-approved drugs for alcohol dependence (disulfiram, naltrexone, and acamprosate) (Johnson et al., 2007). Disulfiram blocks the enzyme acetaldehyde dehydrogenase, preventing formation of acetic acid from acetaldehyde (Heilig and Egli, 2006). Naltrexone blocks mu opioid receptors thought to be responsible for the rewarding effects of alcohol, thereby decreasing alcohol craving (Bouza et al., 2004); however, naltrexone was only effective in a subpopulation of recovering alcoholics. Acamprosate is thought to act by decreasing glutamatergic activity, reducing the negative effects associated with alcohol withdrawal. Treatment options are varied but limited, and there is a high rate of relapse for all existing treatments.

Current evidence suggests that brain immune or pro-inflammatory signaling is linked to alcohol action (Crews, 2012; Mayfield et al., 2013). Long-lasting increases in levels of several cytokines in mouse brain, including monocyte chemoattractant protein-1, MCP-1 (also known as chemokine C-C motif ligand 2, CCL2), were observed after pretreatment with high doses of ethanol followed by injection of lipopolysaccharide (LPS) (Qin et al., 2008). Interestingly, a similar increase in MCP-1 (CCL2) was found in brains of human alcoholics (He and Crews, 2008), and deletion of *Ccl2* or its receptor decreased alcohol consumption in mice (Blednov et al., 2005). Knockout of immune/inflammatory genes in mice, which were selected based on meta-analyses of genes and functional pathways involved in regulation of alcohol drinking in mice (Mulligan et al., 2006), rats (Kimpel et al., 2007), or humans (Liu et al., 2006, 2007; Flatscher-Bader et al., 2008), reduced ethanol intake and preference, further supporting immune pathways in regulating alcohol-mediated behaviors (Blednov et al., 2012). Our research suggests that cytokines (perhaps via endotoxins) promote persistent and excessive alcohol consumption, which may in turn promote further inflammatory responses, producing a positive feedback loop leading to excessive alcohol consumption (Blednov et al., 2011). Furthermore, even a single injection of LPS induces a long-lasting decrease in dopamine neuron firing in the ventral tegmental area, suggesting that cytokine signaling can regulate reward circuitry (Blednov et al., 2011). It is also noteworthy that chronic alcohol consumption and LPS treatment can produce similar changes in mouse brain transcriptomes (Osterndorff-Kahanek et al., 2013).

Phosphodiesterases (PDEs) are a superfamily of enzymes catalyzing the hydrolysis of 3',5'-cyclic adenosine monophosphate (cAMP) and 3',5'-cyclic guanosine monophosphate (cGMP) to their inactive 5'-AMP and 5'-GMP forms, respectively. Increased cAMP and/or cGMP, resulting from inhibition of PDE, reduces inflammatory signaling (Page and Spina, 2011; Jin et al., 2012). Cyclic nucleotides are known to play pivotal roles in a number of cellular functions, including immune and inflammatory responses, cardiac activities, smooth muscle relaxation, depression and cognition. The anti-inflammatory actions of PDE inhibitors are beneficial in treating chronic obstructive pulmonary disease and asthma (Jin et al., 2012; Keravis and Lugnier, 2012). PDEs are encoded by 21 genes, grouped into 11 families, according to their structural similarity. Each gene encodes multiple protein products generated by alternative splicing and/or multiple promoters, resulting in more than 50 different PDE proteins that may be produced in mammalian cells (Bender and Beavo, 2006).

Rolipram, an inhibitor of PDE4, markedly reduced alcohol intake and preference in mice (Hu et al., 2011) and reduced alcohol seeking and consumption in alcohol-preferring fawn-hooded rats (Wen et al., 2012). Rolipram also reduced cocaine conditioned place preference and self-administration (Knapp et al., 1999; Thompson et al., 2004) and inhibited neuroimmune signaling (Zhu et al., 2001). Rolipram and other PDE4 inhibitors have been investigated as potential therapeutics in diverse CNS disease models, including depression, anxiety, schizophrenia, Parkinson's, and Alzheimer's disease (Halene and Siegel, 2007; Kanes et al., 2007; Smith et al., 2009). In this study, we compared the effects of various PDE inhibitors on alcohol consumption and preference in mice. We studied nine PDE inhibitors with different selectivity profiles (Shahid et al., 1991; Raeburn and Karlsson, 1993; Ashton et al., 1994; Meskini et al., 1994; Sugioka et al., 1994; Perry et al., 1998; Dinter et al., 2000): propentofylline (nonspecific); vinpocetine (PDE1); olprinone, milrinone (PDE3); zaprinast (PDE5); rolipram, mesopram, piclamilast, and CDP840 (PDE4) and examined their effects in two different ethanol consumption tests.

## Materials and methods

### Mice

Male C57BL/6J mice were taken from a colony maintained at The University of Texas at Austin (original breeders were purchased from Jackson Laboratories, Bar Harbor, ME). Mice were group-housed four or five to a cage. Food and water were available *ad libitum*. The vivarium was maintained on a 12:12 h light/dark cycle with lights on at 7 a.m. The temperature and humidity of the room were kept constant. Behavioral testing began when the mice were at least 2 months old. All experiments were conducted in isolated behavioral testing rooms in the Animal Resources Center at The University of Texas with reversed light cycle to avoid external distractions. Before beginning experiments, mice were moved to their experimental room and remained there for at least 2 weeks to adapt to the new light cycle. All experiments were approved by the Institutional Animal Care and Use Committee at The University of Texas at Austin.

### Baseline drinking

In both ethanol consumption tests (described below), mice first consumed 15% ethanol for at least 3 weeks. After this period, ethanol consumption was measured for at least 4 days to ensure stable consumption. The criterion for stable consumption was similar values for days 1–2 and days 3–4. For the 24-h two-bottle choice test, ethanol intake was then measured after saline injection for 2 days, and mice were grouped to provide similar levels of ethanol intake and preference based on the first 6 h of consumption during these 2 days. Ethanol and total fluid intake were presented as g/kg/6 h. Measurements made after the next 18 h were presented as percent of corresponding control (values obtained after the next 18 h during the first 2 days of saline injection). In the drinking in the dark (DID) test, mice were grouped to provide similar levels of ethanol intake and preference based on 3 h of consumption during the first 2 days of saline injections. Ethanol and total fluid intake were presented as g/kg/3 h. From day 3, mice were injected once daily with either saline or drugs for up to 10 days, depending on the drug.

### Drug administration

Propentofylline (5 mg/kg), vinpocetine (10 mg/kg), and piclamilast (1 mg/kg) were injected i.p. Olprinone (0.5 mg/kg), milrinone (0.5 mg/kg), mesopram (5 mg/kg), rolipram (1 and 5 mg/kg), CDP840 (10 and 25 mg/kg), and zaprinast (10 mg/kg) were administered p.o. Drugs were purchased from Sigma-Aldrich (St. Louis, MO) or Tocris Bioscience (Minneapolis, MN). All drugs were freshly prepared as suspensions in saline with 4–5 drops of Tween-80 and injected in a volume of 0.05 ml/10 g of body weight 30–60 min before drinking experiments. Saline containing 4–5 drops of Tween-80 was injected in the same volume for control groups. Doses of drugs and routes of administration were based on published data showing anti-inflammatory activity *in vivo* (Yamada et al., 1998; Dinter et al., 2000; Prickaerts et al., 2004; Di Paola et al., 2011; Xiao et al., 2011; Hotte et al., 2012). If the drug was not effective within 4 days, a higher dose was then tested.

### Ethanol drinking—24-h two-bottle choice

The two-bottle choice protocol was carried out as previously described (Blednov et al., 2001). Bottles were weighed twice daily (see below). Food was available *ad libitum*, and mice were weighed every 4 days beginning on day 1. Bottle positions were changed daily to control for position preferences. Ethanol consumption (g/kg body weight/time) was calculated for each mouse. Ethanol (15%) was used in all experiments, and mice had unlimited access to one bottle of water. Ethanol intake was measured 6 h after the beginning of the drinking test and again after the next 18 h. Measurements of ethanol intake, preference, and total fluid intake were averaged over 2 days with different bottle positions. Each point in the graphs (e.g., days 2, 4, 6, 8, 10) represents the average of 2 days of measurement. For example, day 2 is the average of days 1–2 and day 4 is the average of days 3–4.

### Ethanol drinking—limited access in the dark phase (two-bottle choice did)

This was similar to the one-bottle DID test previously described (Rhodes et al., 2005) except that two bottles containing either 15% ethanol or water were used (Blednov and Harris, 2008). The ethanol and water bottles remained in place for 3 h. After their removal, mice had unlimited access to one bottle of water. Bottle positions during 3-h access were changed daily to control for potential side preferences. The ethanol and water bottles were weighed before placing and after removal from experimental cages. Drinking began 3 h after lights off and continued for 3 h. Measurements of ethanol intake, preference, and total fluid intake were averaged over 2 days with different bottle positions. Each point in the graphs (e.g., days 2, 4, 6, 8, 10) represents the average of 2 days of measurement.

### Statistical analysis

Data are reported as the mean ± s.e.m. The statistics software program GraphPad Prism (Jandel Scientific, Costa Madre, CA) was used throughout. Two-Way repeated measures ANOVA with *post-hoc* Bonferroni corrections and Student's *t*-tests were performed.

## Results

### Two-bottle choice with continuous access to alcohol

**Figure 1:**
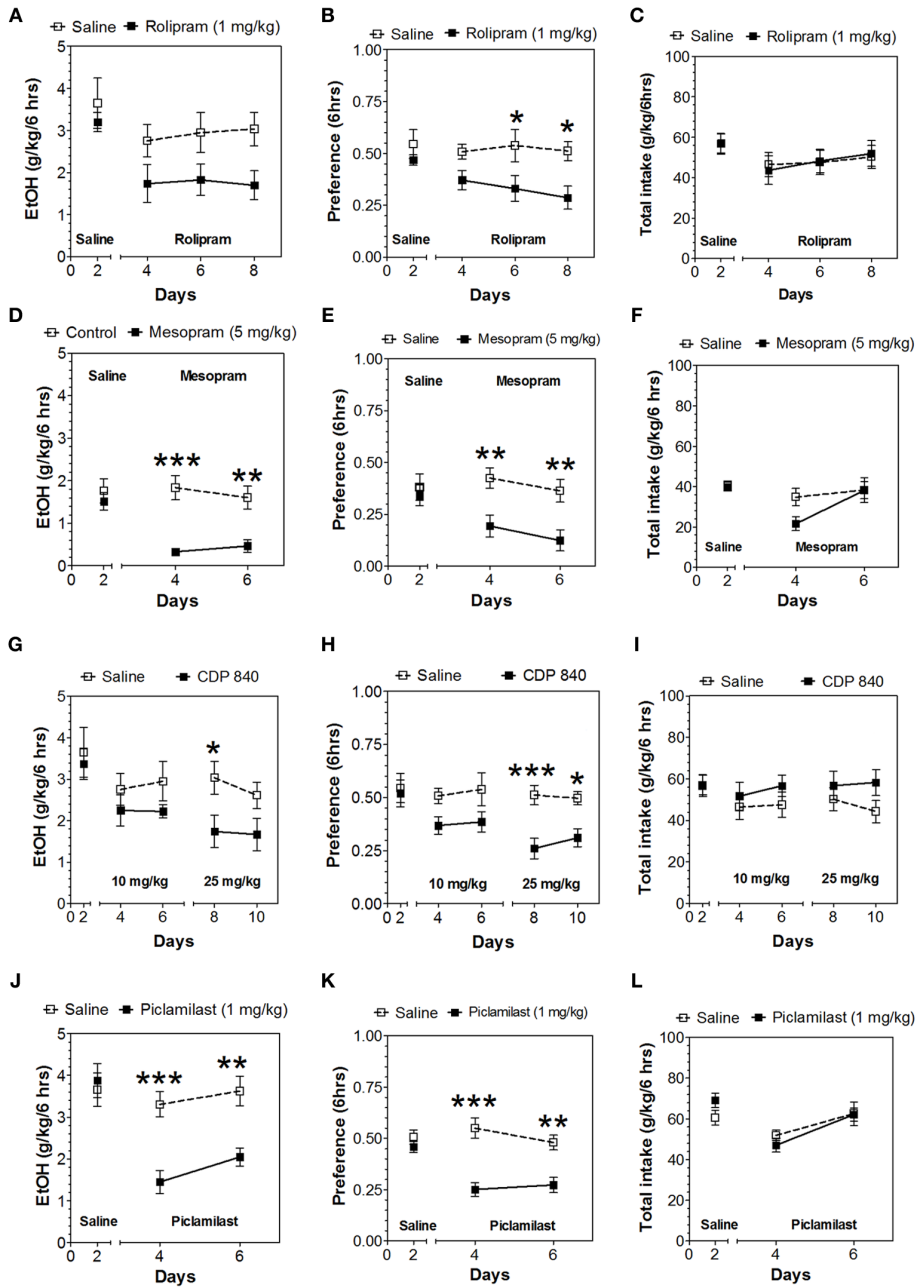
**PDE4 inhibitors reduce alcohol intake during the first 6 h of the 24-h two-bottle choice paradigm**. Ethanol consumption is presented as g/kg/6 h: **(A)** Rolipram **(D)** Mesopram **(G)** CDP840 **(J)** Piclamilast. Preference for ethanol: **(B)** Rolipram **(E)** Mesopram **(H)** CDP840 **(K)** Piclamilast. Total fluid intake (g/kg/6 h): **(C)** Rolipram **(F)** Mesopram **(I)** CDP840 **(L)** Piclamilast. Data were analyzed by Two-Way ANOVA with repeated measures followed by Bonferroni's test for multiple comparisons. ^*^p < 0.05; ^**^p < 0.01; ^***^p < 0.001 compared to control (*n* = 6 per group; EtOH = ethanol).

**Figure 2:**
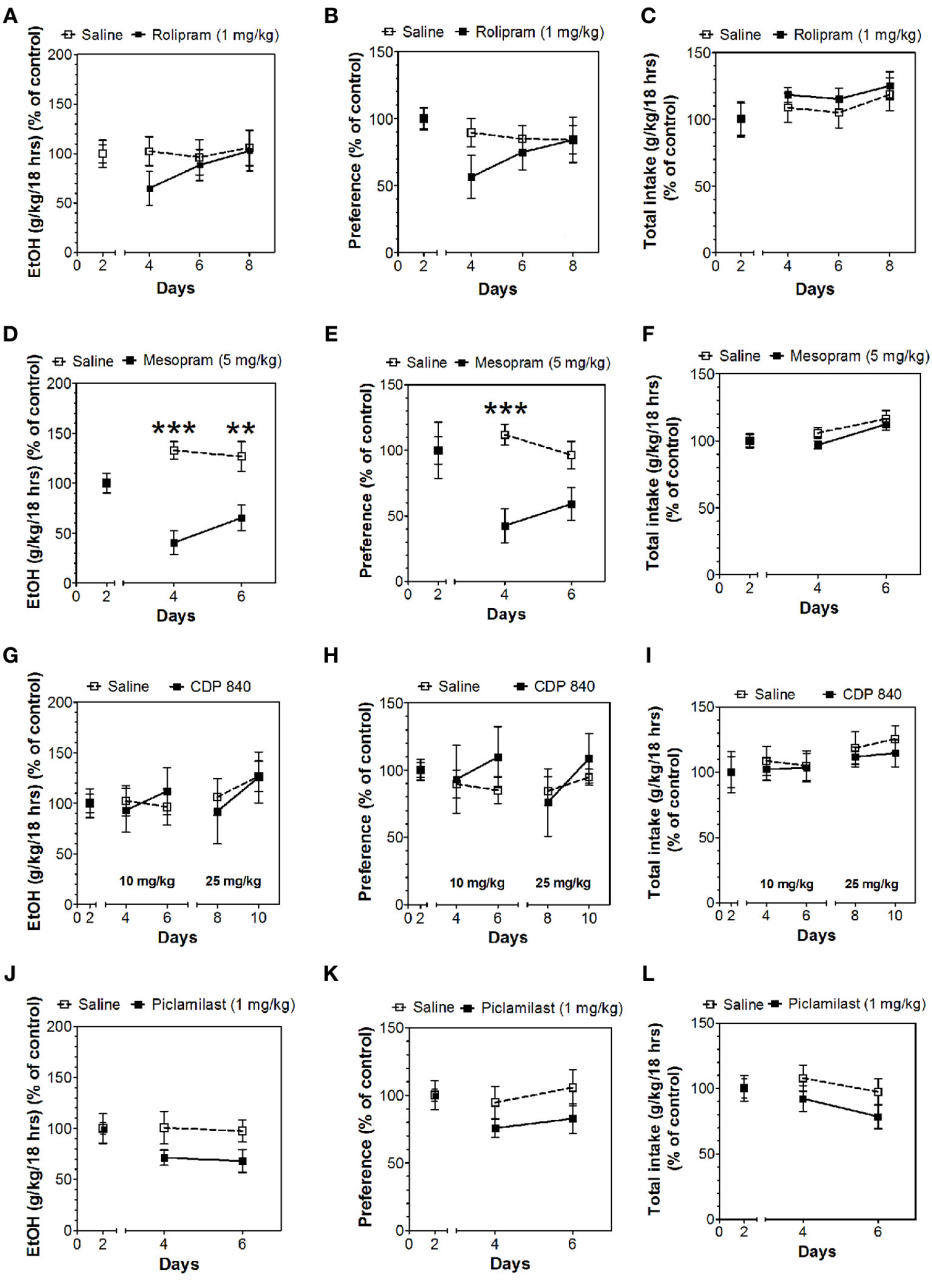
**Effects of PDE4 inhibitors on alcohol intake during the next 18 h of the 24-h two-bottle choice paradigm**. Ethanol consumption is presented as g/kg/18 h: **(A)** Rolipram **(D)** Mesopram **(G)** CDP840 **(J)** Piclamilast. Preference for ethanol: **(B)** Rolipram **(E)** Mesopram **(H)** CDP840 **(K)** Piclamilast. Total fluid intake (g/kg/18 h): **(C)** Rolipram **(F)** Mesopram **(I)** CDP840 **(L)** Piclamilast. Data were analyzed by Two-Way ANOVA with repeated measures followed by Bonferroni's test for multiple comparisons. ^**^p < 0.01; ^***^p < 0.001 compared to control (*n* = 6 per group; EtOH = ethanol).

In the 24-h two-bottle choice continuous access test, all four PDE4 inhibitors (rolipram, mesopram, piclamilast, CDP840) reduced alcohol intake and preference after the first 6 h without affecting total fluid intake (Figure 1; for complete statistical analyses see Data Sheet 1). For rolipram, piclamilast, and CDP840, this effect was not long lasting and was not observed after the next 18 h of ethanol consumption (Figure 2; Data Sheet 2). Mesopram, however, produced a long-lasting reduction of ethanol intake and preference (measured after the next 18 h of ethanol consumption) (Figures 2D,E; Data Sheet 2).

**Figure 3:**
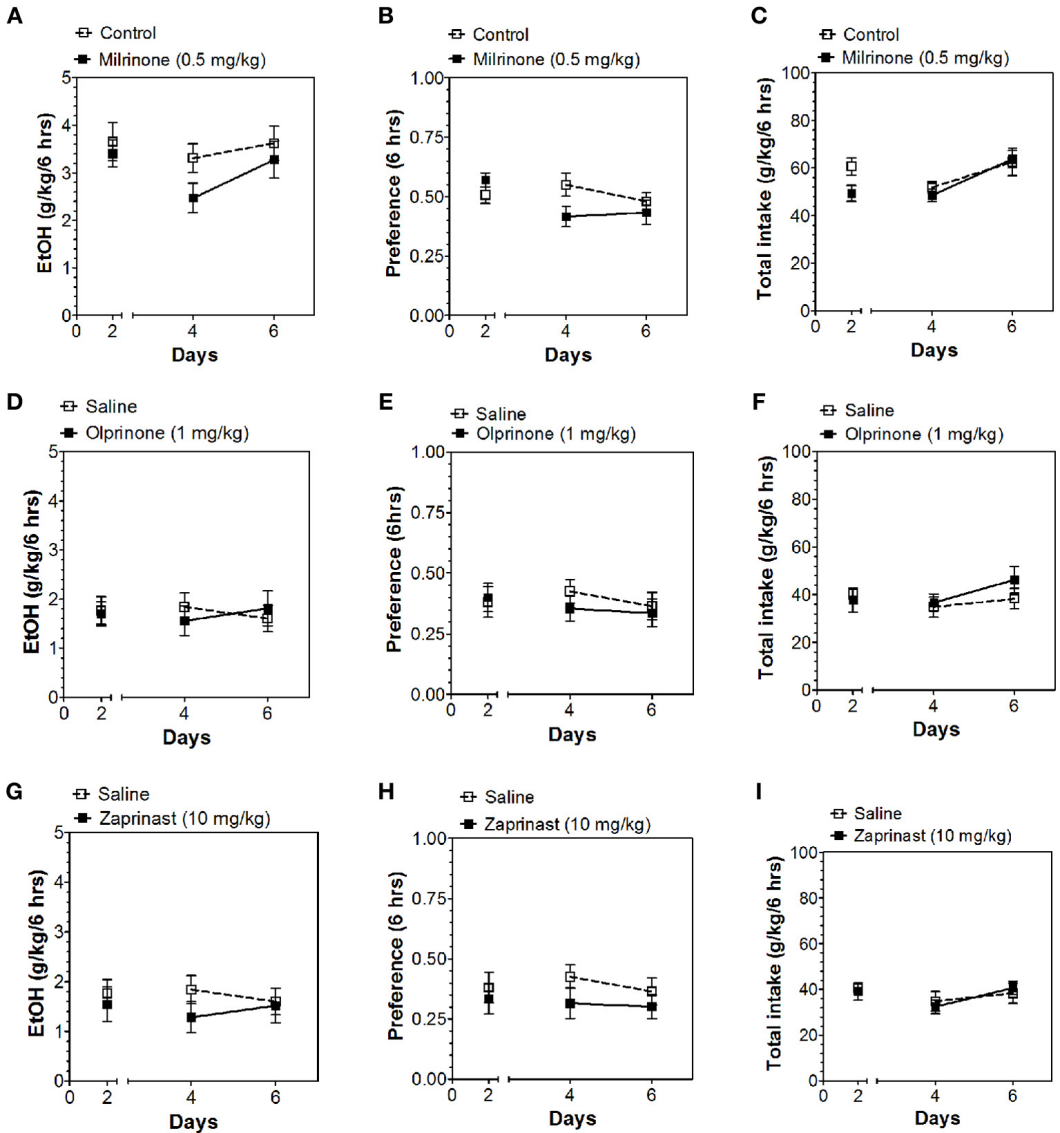
**PDE3 and PDE5 inhibitors do not alter alcohol intake during the first 6 h of the 24-h two-bottle choice paradigm**. Ethanol consumption is presented as g/kg/6 h: **(A)** Milrinone **(D)** Olprinone **(G)** Zaprinast. Preference for ethanol: **(B)** Milrinone **(E)** Olprinone **(H)** Zaprinast. Total fluid intake (g/kg/6 h): **(C)** Milrinone **(F)** Olprinone **(I)** Zaprinast. Data were analyzed by Two-Way ANOVA with repeated measures followed by Bonferroni's test for multiple comparisons (*n* = 6 per group; EtOH = ethanol).

**Figure 4:**
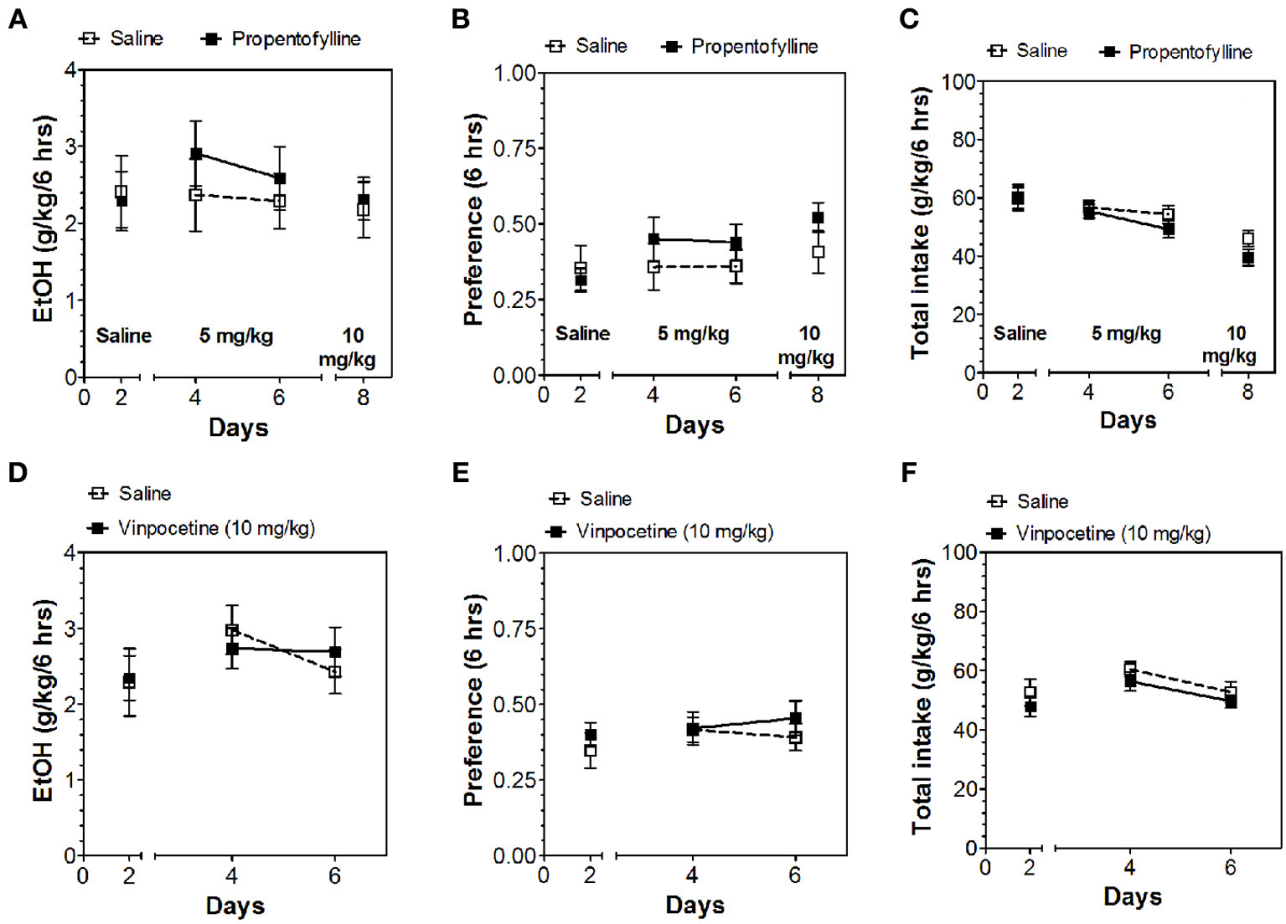
**PDE1 inhibitor vinpocetine and nonspecific inhibitor propentofylline do not alter alcohol intake during the first 6 h of the 24-h two-bottle choice paradigm**. Ethanol consumption is presented as g/kg/6 h: **(A)** Propentofylline **(D)** Vinpocetine. Preference for ethanol: **(B)** Propentofylline **(E)** Vinpocetine. Total fluid intake (g/kg/6 h): **(C)** Propentofylline **(F)** Vinpocetine. Data were analyzed by Two-Way ANOVA with repeated measures followed by Bonferroni's test for multiple comparisons (*n* = 6 per group; EtOH = ethanol).

**Figure 5:**
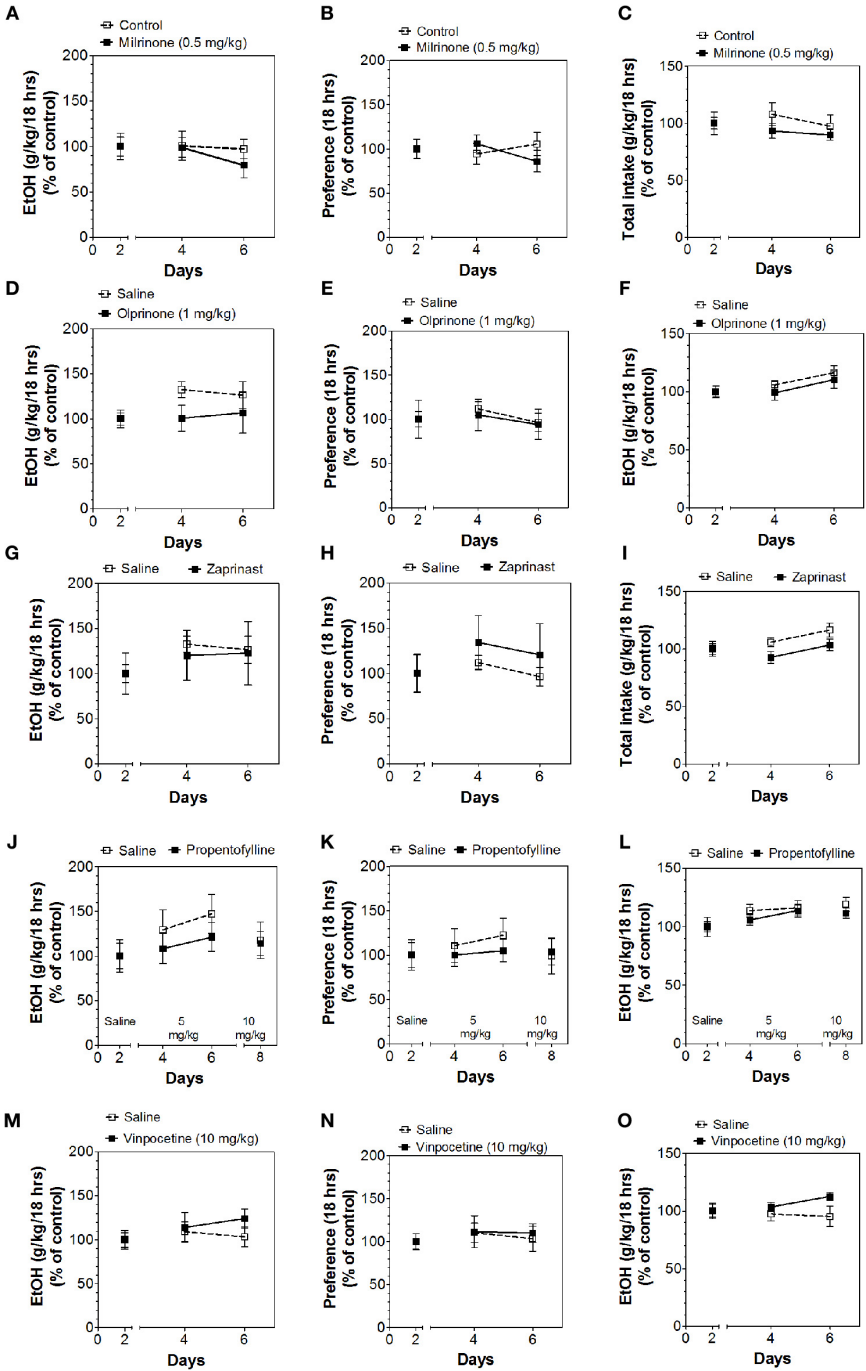
**PDE1,3,5 and nonspecific inhibitors do not alter alcohol intake during the last 18 h of the 24-h two-bottle choice paradigm**. Ethanol consumption is presented as g/kg/18 h: **(A)** Milrinone **(D)** Olprinone **(G)** Zaprinast **(J)** Propentofylline **(M)** Vinpocetine. Preference for ethanol: **(B)** Milrinone **(E)** Olprinone **(H)** Zaprinast **(K)** Propentofylline **(N)** Vinpocetine. Total fluid intake (g/kg/18 h): **(C)** Milrinone **(F)** Olprinone **(I)** Zaprinast **(L)** Propentofylline **(O)** Vinpocetine. Data were analyzed by Two-Way ANOVA with repeated measures followed by Bonferroni's test for multiple comparisons (*n* = 6 per group; EtOH = ethanol).

Inhibitors of other classes of PDEs (olprinone, milrinone, zaprinast, propentofylline, and vinpocetine) had no effect on ethanol consumption in the continuous access test at either time point (Figures 3–5; Data Sheets 3, 4).

### Two-bottle choice with limited access to alcohol

**Figure 6:**
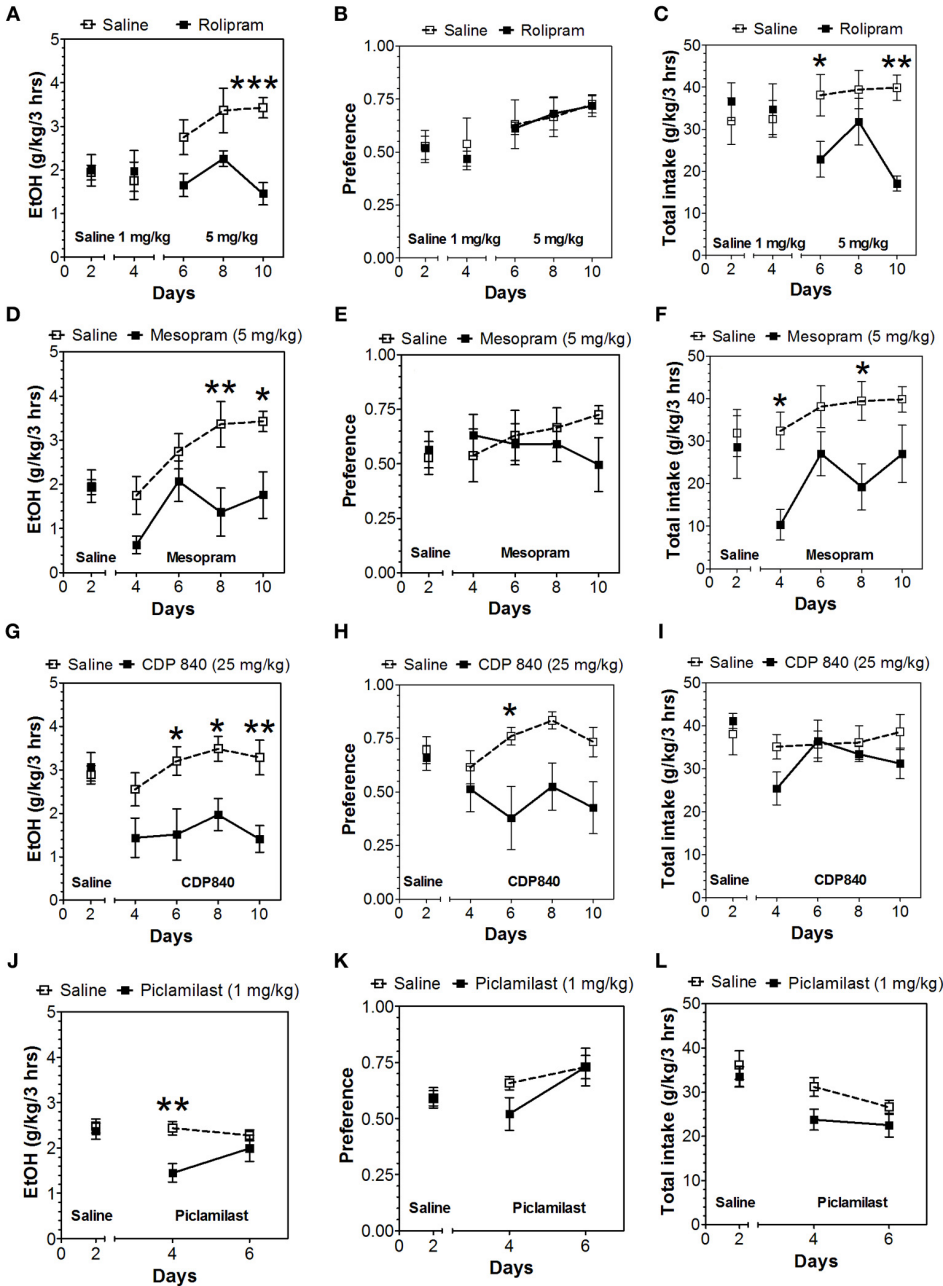
**Effects of PDE4 inhibitors on alcohol intake during a limited access (3-h) two-bottle choice Drinking in the Dark test**. Ethanol consumption is presented as g/kg/3 h: **(A)** Rolipram **(D)** Mesopram **(G)** CDP840 **(J)** Piclamilast. Preference for ethanol: **(B)** Rolipram **(E)** Mesopram **(H)** CDP840 **(K)** Piclamilast. Total fluid intake (g/kg/3 h): **(C)** Rolipram **(F)** Mesopram **(I)** CDP840 **(L)** Piclamilast. Data were analyzed by Two-Way ANOVA with repeated measures followed by Bonferroni's test for multiple comparisons. ^*^p < 0.05; ^**^p < 0.01; ^***^p < 0.001 compared to control (*n* = 6 per group; EtOH = ethanol).

Because of the selective reduction by PDE4 inhibitors in continuous access drinking, only this subtype was studied in the limited access test. In contrast to continuous access, rolipam (1 mg/kg) did not change consumption in limited access (Figures 6A–C; Data Sheet 5); however, 5 mg/kg rolipram as well as mesopram, piclamilast, and CDP840 strongly reduced alcohol intake (Figures 6A,D,G,J; Data Sheet 5). Of these inhibitors, CDP840 also reduced alcohol preference (Figures 6H,I), whereas rolipram, mesopram, and piclamilast failed to reduce alcohol preference (Figures 6B,E,K) because they reduced total fluid intake (Figures 6C,F,L; Data Sheet 5).

## Discussion

Rolipram was previously shown to reduce ethanol intake and preference in mice (Hu et al., 2011) and rats (Wen et al., 2012) in 24-h two-bottle choice tests, which is consistent with our results in mice. We further demonstrate that other selective PDE4 inhibitors (but not other classes of PDE inhibitors) reduce intake and preference in the 24-h two-bottle choice paradigm. Like rolipram, CDP840 and piclamilast transiently reduce intake, whereas mesopram produces long-lasting decreases. Hu et al. (2011) did not report transient effects of rolipram, but they injected it i.p. twice daily, whereas we used a once daily oral administration. CDP840 also reduces ethanol intake and preference in the limited access test, whereas the other PDE4 inhibitors do not alter preference. Reduction of ethanol and total fluid intake, without changes in preference, in the limited access test may be due to the sedative activity of PDE4 inhibitors (Hu et al., 2011). Any sedative effects would be likely to interfere with drinking in this limited 3-h time period. However, mice in limited access or “binge” models may also display a different sensitivity to pharmacological intervention compared to mice undergoing continuous access (Crabbe et al., 2011). Furthermore, these two treatment paradigms induce different transcriptome signatures in mouse brain (Osterndorff-Kahanek et al., 2013). Other classes of inhibitors (nonspecific or PDE1, 3, or 5 inhibitors) had no effect on alcohol consumption in the continuous access test. Similar to our results with propentofylline, ibudilast (another nonspecific PDE inhibitor) did not change alcohol intake in non-dependent mice; however, ibudilast reduced drinking in alcohol-dependent mice and in alcohol-preferring P and HAD1 rats (Bell et al., 2013). Also, mice with genetic deletion of both adenylyl cyclase 1 (AC1) and AC8 exhibited decreased ethanol consumption and enhanced sensitivity to ethanol sedation (Maas et al., 2005). In contrast, mice lacking AC5 showed increased ethanol consumption and preference and reduced sensitivity to ethanol sedation (Kim et al., 2011).

It is important to note that the PDE inhibitors which did not alter alcohol consumption are nevertheless capable of exerting other CNS-dependent effects on behavior and neurophysiology. For example, olprinone and milrinone provided significant neuroprotection after cerebral ischemia/reperfusion injury in rats (Saklani et al., 2010; Genovese et al., 2011). Zaprinast and other PDE5 inhibitors improved memory (Prickaerts et al., 2004), and propentofylline improved learning and memory deficits induced by beta-amyloid protein (Yamada et al., 1998). Also, vinpocetine ameliorated hyperactivity in a mouse model of fetal alcohol spectrum disorder (Nunes et al., 2011).

Despite a long history of availability (since the late 1980's), PDE inhibitors are of limited clinical use because of lack of efficacy and/or gastroenterological side effects such as nausea and vomiting (Giembycz, 2008). One approach to mitigate the adverse effects is seeking agents with selectivity for PDE4 isoforms devoid of these effects. PDE4 isoenzymes are encoded by four genes (A, B, C, D), and it is believed that inhibition of PDE4D promotes emesis. Selective inhibition of PDE4A and/or PDE4B in pro-inflammatory and immune cells is believed to produce desired anti-inflammatory effects (Giembycz, 2008; Jin et al., 2012). PDE4B is of particular interest because it is localized in brain reward pathways (Cherry and Davis, 1999), and its expression is upregulated in mice with a genetic predisposition for high alcohol consumption (Mulligan et al., 2006). Rolipram inhibits PDE4 catalytic activity with micromolar potency but also binds to a high affinity (nanomolar) site on PDE4 (Schneider et al., 1986). Duplantier et al. (1996) noted a relationship between the high affinity site (in rats) and emesis (in ferrets), and there is a correlation between high affinity binding of denbufylline, rolipram, piclamilast, CDP840, and KF19514 and their emetic activity in the house musk shrew (Hirose et al., 2007). Thus, inhibitors with lower selectivity for PDE4D and lower binding to the high affinity site may reduce the side effects of these drugs.

Given the proposed link between neuroimmune signaling and alcohol consumption and dependence (Harris and Blednov, 2013; Mayfield et al., 2013), PDE inhibitors could potentially decrease alcohol consumption by inhibiting inflammatory signaling. Indeed, rolipram dose-dependently reduced LPS-induced production of TNFα in rat brain and microglia (Buttini et al., 1997; Yoshikawa et al., 1999). Furthermore, LPS, pro-inflammatory cytokines, and ethanol all increased the activity of PDE4 in several types of cells (microglia, astrocytes, bronchial epithelial), and these effects were reversed by rolipram (Buttini et al., 1997; Forget et al., 2003; Ghosh et al., 2012).

Our work represents the first comparison of different classes of PDE inhibitors in different ethanol drinking tests in mice with a genetic predisposition for high alcohol consumption. We provide strong evidence for a selective role of PDE4 inhibitors in reducing alcohol consumption and preference. PDE4 inhibitors have also been investigated in animal models of other CNS disorders, and the availability of more selective isoforms devoid of side effects may prove beneficial in the treatment of alcohol abuse and other inflammatory diseases.

In addition to behavioral evidence for specific PDEs in alcohol drinking in rodents, there is evidence for altered expression of PDE genes in mice following chronic ethanol. For example, *Pde10* and *Pde1b* were upregulated in mouse prefrontal cortex after chronic ethanol intake (Osterndorff-Kahanek et al., 2013), and *Pde4b* was upregulated while *Pde10a* was downregulated in brains of mice showing a genetic predisposition to high alcohol consumption (Mulligan et al., 2006). Furthermore, the Collaborative Study on the Genetics of Alcoholism (COGA) identified a candidate single nucleotide polymorphism near *PDE11A* based on differing allele frequencies between alcohol-dependent and control individuals (Johnson et al., 2006). While both mouse and human studies nominate PDE genes, confirmation using multiple datasets combined with behavioral validation will ultimately be important to link them to addiction vulnerability.

## Author contributions

Yuri A. Blednov designed and performed experiments, analyzed data, and wrote the paper; Jillian M. Benavidez and Mendy Black performed experiments; R. Adron Harris designed experiments and wrote the paper.

### Conflict of interest statement

The authors declare that the research was conducted in the absence of any commercial or financial relationships that could be construed as a potential conflict of interest.

## Abbreviations

PDE: phosphodiesterases
cAMP: 3',5'-cyclic adenosine monophosphate
cGMP: 3',5'-cyclic guanosine monophosphate.
