# Supplementary material for: Inhibition of phosphodiesterase 4 reduces ethanol intake and preference in C57BL/6J mice
Source: Front Neurosci. 2014 May 27;8:129. doi: 10.3389/fnins.2014.00129 (PMC4034339; doi:10.3389/fnins.2014.00129)
Supplement: Supplementary file 1 [file DataSheet1.PDF]

Data Sheet 1. Statistical analyses of the effects of PDE4 inhibitors on alcohol intake after the first 6 hours in the two-bottle choice test.

Drug	Dose	Factors	Ethanol consumption		
			Amount of ethanol consumed (g/kg/6 hr)	Preference	Total fluid intake (g/kg/6 hr)
Rolipram	1 mg/kg	treatment	**F(1,10)=5.7;p<0.05**	**F(1,10)=8.2;p<0.05**	F(1,10)=0.1;p>0.05
		time	F(2,20)=0.2;p>0.05	F(2,20)=0.7;p>0.05	F(2,20)=1.8;p>0.05
		interaction	F(2,20)=0.2;p>0.05	F(2,20)=0.8;p>0.05	F(2,20)=0.3;p>0.05
Mesopram	5 mg/kg	treatment	**F(1,12)=24.7;p<0.001**	**F(1,12)=13.1;p<0.01**	F(1,12)=1.2;p>0.05
		time	F(1,12)=0.1;p>0.05	F(1,12)=3.7;p>0.05	**F(1,12)=13.9;p<0.01**
		interaction	F(1,12)=1.9;p>0.05	F(1,12)=0.1;p>0.05	**F(1,12)=6.0;p<0.05**
Piclamilast	1 mg/kg	treatment	**F(1,10)=20.1;p<0.01**	**F(1,10)=26.6;p<0.001**	F(1,10)=0.6;p>0.05
		time	**F(1,10)=9.2;p<0.05**	F(1,10)=0.8;p>0.05	**F(1,10)=7.9;p<0.05**
		interaction	F(1,10)=0.9;p>0.05	F(1,10)=2.9;p>0.05	F(1,10)=0.2;p>0.05
CDP840	10 mg/kg	treatment	F(1,10)=1.9;p>0.05	**F(1,10)=5.3;p<0.05**	F(1,10)=0.8;p>0.05
		time	F(1,10)=0.1;p>0.05	F(1,10)=0.3;p>0.05	F(1,10)=0.9;p>0.05
		interaction	F(1,10)=0.2;p>0.05	F(1,10)=0.1;p>0.05	F(1,10)=0.4;p>0.05
	25 mg/kg	treatment	**F(1,10)=5.0;p<0.05**	**F(1,10)=19.4;p<0.01**	F(1,10)=1.5;p>0.05
		time	F(1,10)=1.9;p>0.05	F(1,10)=0.3;p>0.05	F(1,10)=1.0;p>0.05
		interaction	F(1,10)=0.9;p>0.05	F(1,10)=0.9;p>0.05	F(1,10)=2.9;p>0.05

Statistically significant results are shown in bold font (two-way ANOVA).
