# Supplementary material for: Inhibition of phosphodiesterase 4 reduces ethanol intake and preference in C57BL/6J mice
Source: Front Neurosci. 2014 May 27;8:129. doi: 10.3389/fnins.2014.00129 (PMC4034339; doi:10.3389/fnins.2014.00129)
Supplement: Supplementary file 2 [file DataSheet2.PDF]

Data Sheet 2. Statistical analyses of the effects of PDE4 inhibitors on alcohol intake after the next 18 hours in the two-bottle choice test.

Drug	Dose	Factors	Ethanol consumption		
			Amount of ethanol consumed (g/kg/18 hr)	Preference	Total fluid intake (g/kg/18 hr)
Rolipram	1 mg/kg	treatment	F(1,10)=0.5;p>0.05	F(1,10)=0.8;p>0.05	F(1,10)=0.4;p>0.05
		time	F(2,20)=2.5;p>0.05	F(2,20)=1.1;p>0.05	**F(2,20)=3.9;p<0.05**
		interaction	F(2,20)=2.0;p>0.05	F(2,20)=2.5;p>0.05	F(2,20)=0.1;p>0.05
Mesopram	5 mg/kg	treatment	**F(1,12)=29.9;p<0.001**	**F(1,12)=15.8;p<0.01**	F(1,12)=1.9;p>0.05
		time	F(1,12)=0.8;p>0.05	F(1,12)=0.1;p>0.05	**F(1,12)=9.5;p<0.01**
		interaction	F(1,12)=2.3;p>0.05	F(1,12)=3.7;p>0.05	**F(1,12)=6.0;p<0.05**
Piclamilast	1 mg/kg	treatment	F(1,10)=3.9;p>0.05	F(1,10)=4.1;p>0.05	F(1,10)=1.7;p>0.05
		time	F(1,10)=0.2;p>0.05	F(1,10)=0.6;p>0.05	**F(1,10)=10.6;p<0.01**
		interaction	F(1,10)=0.1;p>0.05	F(1,10)=0.1;p>0.05	F(1,10)=0.2;p>0.05
CDP840	10 mg/kg	treatment	F(1,10)=0.1;p>0.05	F(1,10)=0.3;p>0.05	F(1,10)=0.1;p>0.05
		time	F(1,10)=0.6;p>0.05	F(1,10)=0.5;p>0.05	F(1,10)=0.2;p>0.05
		interaction	F(1,10)=2.4;p>0.05	F(1,10)=1.5;p>0.05	F(1,10)=0.6;p>0.05
	25 mg/kg	treatment	F(1,10)=0.1;p>0.05	F(1,10)=0.1;p>0.05	F(1,10)=0.4;p>0.05
		time	**F(1,10)=7.8;p<0.05**	F(1,10)=4.3;p>0.05	F(1,10)=1.9;p>0.05
		interaction	F(1,10)=0.4;p>0.05	F(1,10)=1.2;p>0.05	F(1,10)=0.3;p>0.05

Statistically significant results are shown in bold font (two-way ANOVA).
