# Supplementary material for: Inhibition of phosphodiesterase 4 reduces ethanol intake and preference in C57BL/6J mice
Source: Front Neurosci. 2014 May 27;8:129. doi: 10.3389/fnins.2014.00129 (PMC4034339; doi:10.3389/fnins.2014.00129)
Supplement: Supplementary file 3 [file DataSheet3.PDF]

Data Sheet 3. Statistical analyses of the effects of PDE inhibitors on alcohol intake after the first 6 hours in the two-bottle choice test.

Drug	Dose	Factors	Ethanol consumption		
			Amount of ethanol consumed (g/kg/6 hr)	Preference	Total fluid intake (g/kg/6 hr)
Milrinone	0.5 mg/kg	treatment	F(1,10)=1.7;p>0.05	F(1,10)=2.5;p>0.05	F(1,10)=0.1;p>0.05
		time	**F(1,10)=9.2;p<0.05**	F(1,10)=0.9;p>0.05	**F(1,10)=10.3;p<0.01**
		interaction	F(1,10)=1.7;p>0.05	F(1,10)=2.5;p>0.05	F(1,10)=0.3;p>0.05
Olprinone	1 mg/kg	treatment	F(1,12)=0.1;p>0.05	F(1,12)=0.8;p>0.05	F(1,12)=0.8;p>0.05
		time	F(1,12)=0.1;p>0.05	F(1,12)=7.7;p>0.05	F(1,12)=4.2;p>0.05
		interaction	F(1,12)=2.2;p>0.05	F(1,12)=0.2;p>0.05	F(1,12)=0.9;p>0.05
Zaprinast	10 mg/kg	treatment	F(1,12)=0.7;p>0.05	F(1,12)=2.0;p>0.05	F(1,12)=0.1;p>0.05
		time	F(1,12)=0.1;p>0.05	F(1,12)=0.6;p>0.05	**F(1,12)=7.9;p<0.05**
		interaction	F(1,12)=1.2;p>0.05	F(1,12)=0.2;p>0.05	F(1,12)=1.3;p>0.05
Propentofylline	5 mg/kg	treatment	F(1,17)=0.6;p>0.05	F(1,17)=1.0;p>0.05	F(1,17)=0.8;p>0.05
		time	F(1,17)=0.9;p>0.05	F(1,17)=0.1;p>0.05	**F(1,17)=5.0;p<0.05**
		interaction	F(1,17)=0.3;p>0.05	F(1,17)=0.1;p>0.05	F(1,17)=1.1;p>0.05
	10 mg/kg	Student's t-test	p>0.05	p>0.05	p>0.05
Vinpocetine	10 mg/kg	treatment	F(1,16)=0.1;p>0.05	F(1,16)=0.3;p>0.05	F(1,16)=1.0;p>0.05
		time	**F(1,16)=4.9;p<0.05**	F(1,16)=0.1;p>0.05	**F(1,16)=12.9;p<0.05**
		interaction	F(1,16)=0.9;p>0.05	F(1,16)=2.2;p>0.05	F(1,16)=0.1;p>0.05

Statistically significant results are shown in bold font (two-way ANOVA or Student's t-test).
