# Supplementary material for: Inhibition of phosphodiesterase 4 reduces ethanol intake and preference in C57BL/6J mice
Source: Front Neurosci. 2014 May 27;8:129. doi: 10.3389/fnins.2014.00129 (PMC4034339; doi:10.3389/fnins.2014.00129)
Supplement: Supplementary file 4 [file DataSheet4.PDF]

Data Sheet 4. Statistical analyses of the effects of PDE inhibitors on alcohol intake after the next 18 hours in the two-bottle choice test.

Drug	Dose	Factors	Ethanol consumption		
			Amount of ethanol consumed (g/kg/18 hr)	Preference	Total fluid intake (g/kg/18 hr)
Milrinone	0.5 mg/kg	treatment	F(1,10)=0.3;p>0.05	F(1,10)=0.1;p>0.05	F(1,10)=1.1;p>0.05
		time	F(1,10)=1.9;p>0.05	F(1,10)=0.2;p>0.05	F(1,10)=2.3;p>0.05
		interaction	F(1,10)=1.0;p>0.05	F(1,10)=2.9;p>0.05	F(1,10)=0.6;p>0.05
Olprinone	1 mg/kg	treatment	F(1,12)=1.6;p>0.05	F(1,12)=0.1;p>0.05	F(1,12)=0.8;p>0.05
		time	F(1,12)=0.1;p>0.05	F(1,12)=2.5;p>0.05	**F(1,12)=7.3;p<0.05**
		interaction	F(1,12)=0.3;p>0.05	F(1,12)=0.1;p>0.05	F(1,12)=0.1;p>0.05
Zaprinast	10 mg/kg	treatment	F(1,12)=0.1;p>0.05	F(1,12)=0.5;p>0.05	F(1,12)=4.3;p>0.05
		time	F(1,12)=0.1;p>0.05	F(1,12)=4.2;p>0.05	**F(1,12)=9.7;p<0.01**
		interaction	F(1,12)=0.2;p>0.05	F(1,12)=0.1;p>0.05	F(1,12)=0.1;p>0.05
Propentofylline	5 mg/kg	treatment	F(1,17)=0.8;p>0.05	F(1,17)=0.4;p>0.05	F(1,17)=0.5;p>0.05
		time	**F(1,17)=5.8;p<0.05**	F(1,17)=2.7;p>0.05	F(1,17)=3.8;p>0.05
		interaction	F(1,17)=0.2;p>0.05	F(1,17)=0.5;p>0.05	F(1,17)=1.1;p>0.05
	10 mg/kg	Student's t-test	p>0.05	p>0.05	p>0.05
Vinpocetine	10 mg/kg	treatment	F(1,16)=0.6;p>0.05	F(1,16)=0.1;p>0.05	F(1,16)=2.6;p>0.05
		time	F(1,16)=0.1;p>0.05	F(1,16)=0.2;p>0.05	F(1,16)=0.6;p>0.05
		interaction	F(1,16)=1.0;p>0.05	F(1,16)=0.1;p>0.05	F(1,16)=1.6;p>0.05

Statistically significant results are shown in bold font (two-way ANOVA or Student's t-test).
