# Supplementary material for: Inhibition of phosphodiesterase 4 reduces ethanol intake and preference in C57BL/6J mice
Source: Front Neurosci. 2014 May 27;8:129. doi: 10.3389/fnins.2014.00129 (PMC4034339; doi:10.3389/fnins.2014.00129)
Supplement: Supplementary file 5 [file DataSheet5.PDF]

Data Sheet 5. Statistical analyses of the effects of PDE4 inhibitors on alcohol intake in the two-bottle choice drinking in the dark paradigm.

Drug	Dose	Factors	Ethanol consumption		
			Amount of ethanol consumed (g/kg/3 hr)	Preference	Total fluid intake (g/kg/3 hr)
Rolipram	1 mg/kg	Student t-test	$p>0.05$	$p>0.05$	$p>0.05$
	5 mg/kg	treatment	**$F(1,10)=17.3; p<0.01$**	$F(1,10)=0.1; p>0.05$	**$F(1,10)=13.5; p<0.01$**
		time	$F(2,20)=2.5; p>0.05$	$F(2,20)=2.2; p>0.05$	$F(2,20)=2.0; p>0.05$
		interaction	$F(2,20)=1.6; p>0.05$	$F(2,20)=0.1; p>0.05$	$F(2,20)=2.1; p>0.05$
Mesopram	5 mg/kg	treatment	**$F(1,10)=11.2; p<0.01$**	$F(1,10)=0.3; p>0.05$	**$F(1,10)=10.9; p<0.01$**
		time	**$F(3,30)=6.0; p<0.01$**	$F(3,30)=0.1; p>0.05$	**$F(3,30)=4.2; p<0.05$**
		interaction	$F(3,30)=1.2; p>0.05$	$F(3,30)=1.7; p>0.05$	$F(3,30)=1.0; p>0.05$
Piclamilast	1 mg/kg	treatment	**$F(1,14)=7.7; p<0.05$**	$F(1,14)=0.7; p>0.05$	**$F(1,14)=5.7; p<0.05$**
		time	$F(1,14)=1.2; p>0.05$	**$F(1,14)=11.9; p<0.01$**	$F(1,14)=2.1; p>0.05$
		interaction	$F(1,14)=4.1; p>0.05$	$F(1,14)=2.9; p>0.05$	$F(1,14)=0.7; p>0.05$
CDP840	25 mg/kg	treatment	**$F(1,10)=11.2; p<0.01$**	**$F(1,10)=5.8; p<0.05$**	$F(1,10)=1.3; p>0.05$
		time	$F(3,30)=2.6; p>0.05$	$F(3,30)=1.6; p>0.05$	$F(3,30)=2.3; p>0.05$
		interaction	$F(3,30)=0.8; p>0.05$	$F(3,30)=1.9; p>0.05$	$F(3,30)=1.9; p>0.05$

Statistically significant results are shown in bold font (two-way ANOVA or Student's t-test).
